# Altered Norbin Expression in Patients with Epilepsy and a Rat Model

**DOI:** 10.1038/s41598-017-13248-9

**Published:** 2017-10-25

**Authors:** Yali Xu, Zengyou Li, Li Yao, Xingping Zhang, Dan Gan, Manchun Jiang, Na Wang, Guojun Chen, Xuefeng Wang

**Affiliations:** 1 Department of Geriatrics, Chongqing General Hospital, 104 Pipashan Street, Chongqing, China; 2 Department of Neurology, The First Affiliated Hospital, Chongqing Medical University, 1 Youyi Road, Chongqing, China; 3 Health Checkup Center, Chongqing General Hospital, 104 Pipashan Street, Chongqing, China

## Abstract

Norbin is widely distributed in neuronal tissues, is a regulator of Ca2^+^/calmodulin-dependent protein kinase II (CaMKII) phosphorylation. Norbin is also an important endogenous modulator of metabotropic glutamate receptor 5 (mGluR5) signaling, and nervous system-specific homozygous gene disruptions, result in epileptic seizures. In this study, we aimed to investigate norbin expression patterns in epilepsy and to elucidate the relationships between norbin and mGluR5 and p-CaMKII in epilepsy. Double-immunolabeling, immunohistochemistry and immunoblotting studies showed that norbin was downregulated in the temporal neocortex of patients with temporal lobe epilepsy (TLE) compared with control subjects. Moreover, in a rat model of lithium chloride-pilocarpine-induced epilepsy, norbin expression began to decrease at 6 h after the onset of status epilepticus and remained at a low level until 60 days. In addition, p-CaMKII expression was significantly increased in both patients with TLE and in animal model. Norbin and mGluR5 were found to be co-expressed in neurons of epileptic tissues. Finally, norbin over-expression facilitated by injections of adeno-associated viral vector into the rat hippocampus increased latency and survival in the lithium chloride-pilocarpine model. Thus, our results indicate norbin participates in the pathogenesis of epilepsy, perhaps by modulating mGluR5 signaling, regulating CaMKII phosphorylation, and may exert antiepileptic effects.

## Introduction

Norbin, identified in 1997, is a cytoplasmic protein weighing approximately 78 KDa that is widely distributed in neuronal tissues. Norbin promotes neurite outgrowth^1^ and is a regulator of adult hippocampal neurogenesis^2^. Moreover, norbin expression enhances metabotropic glutamate receptor 5 (mGluR5) signaling *in vitro* expression systems, and the neuron-specific protein plays a critical role in mGluR5 localization^3^. Norbin is also a negative regulator of Ca2+/calmodulin-dependent protein kinase II (CaMKII) (Thr-286) phosphorylation and is essential for spatial learning^4^. In addition, nervous system-specific homozygous gene disruptions, which interfere with norbin expression, result in epileptic seizures^4^.

Norbin expression likely plays an important role in epilepsy; however, to our knowledge, no study has addressed the role of norbin in the brain tissues of patients with epilepsy or rat models. In this study, we investigated norbin expression in the temporal neocortex of patients with temporal lobe epilepsy (TLE) and also assessed p-CaMKII expression and mGluR5 signaling in the epileptic brain tissues of patients with epilepsy. To extend the results of the analyses of human tissues, we investigated norbin expression in the hippocampus and adjacent cortical tissues of a rat model of TLE at different time points after the onset of epilepsy. In addition, we investigated p-CaMKII expression in both patients and the animal model and assessed mGluR5 and norbin co-expression after the onset of epilepsy. Moreover, we also observed the influence of norbin over-expression in the rat hippocampus in the lithium chloride-pilocarpine-induced model of acute epilepsy.

## Results

### Norbin neuronal localization and deceased norbin protein expression in the temporal neocortex of patients with TLE

**Figure 1:**
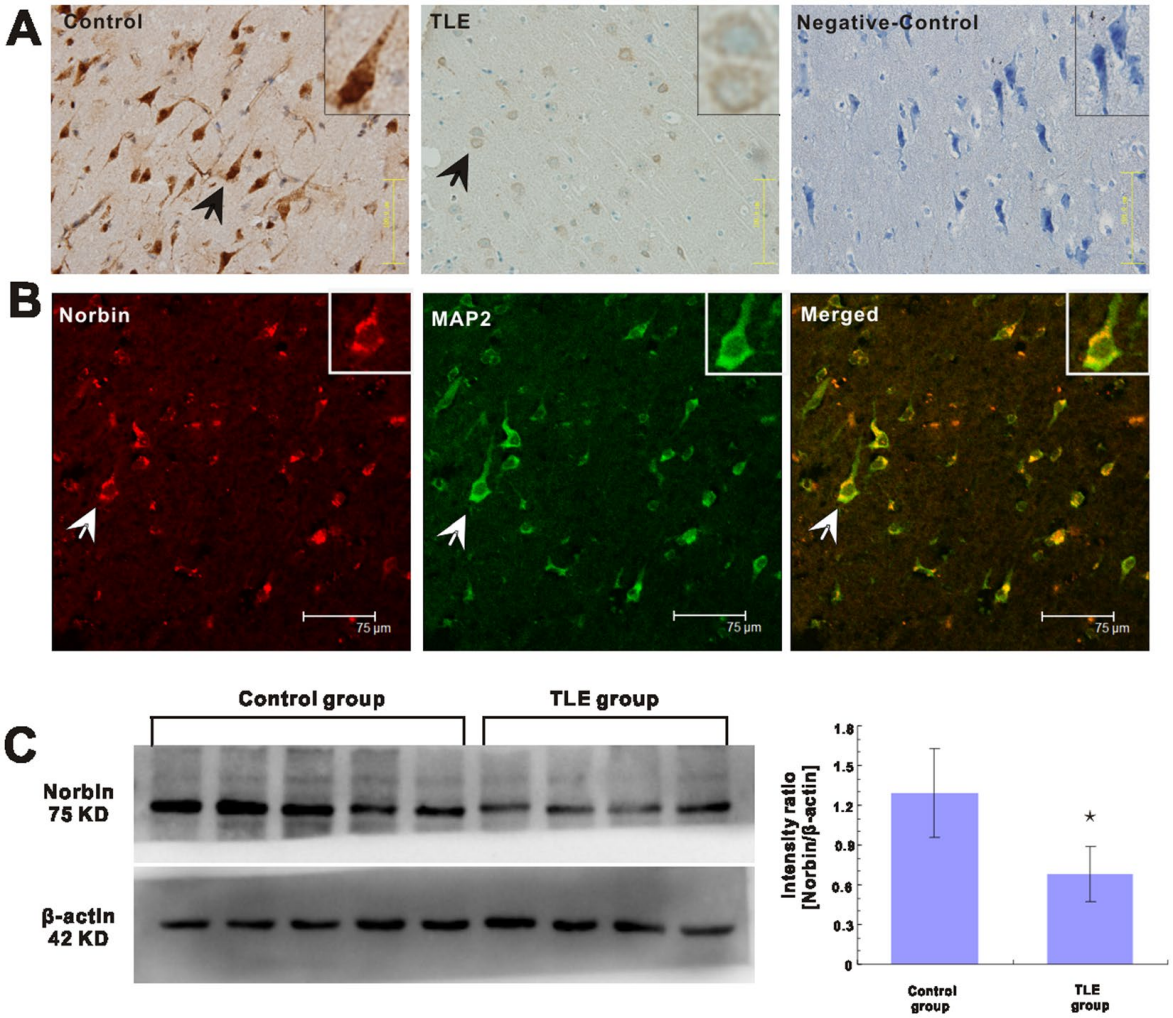
Immunohistochemical, double-immunofluorescence labelling, and western blotting analyses of norbin expression in the temporal neocortex of patients with TLE (n = 32) or without TLE (n = 14). (**A**) Immunohistochemistry analysis of norbin expression in the human temporal neocortex. The arrowheads indicate norbin-positive cells. (Control) Strong norbin immunoreactivity in the temporal neocortex of a control subject. (TLE) Faint norbin immunoreactivity in the temporal neocortex of a patient with TLE. (Negative-control) Secondary antibody-only reactivity control. Insets show the representative morphology at a higher magnification. (**B**) Double-immunofluorescence labelling analysis shows that norbin (red) and MAP2 (green) are coexpressed (merged) in the temporal neocortex of patients with epilepsy. The arrowheads indicate norbin+/MAP2+ cells. (**C**) Western blotting analysis of norbin expression in the temporal neocortex of humans. (Left) Proteins from individual brain homogenates from patients with TLE and control subjects were separated by gradient SDS-PAGE. Immunoreactive staining for norbin was weaker in patients with TLE than in controls. (Right) Comparison of the mean OD value showing that norbin expression levels were significantly lower in patients with TLE than in controls (*p < 0.05).

The norbin protein was found to be expressed in the membrane and cytoplasm of neurons in the temporal neocortical tissues of non-epileptic autopsy controls and patients with TLE. Strong norbin staining was observed in the corresponding sections from subjects in the non-epileptic group, whereas faint immunoreactivity for norbin was observed in the corresponding sections from patients in the TLE group (Fig. 1A). No immunoreactivity was observed in the negative control group, in which the primary antibody had been omitted.

Using double-immunofluorescence labeling, we found that norbin was expressed exclusively in neurons in the temporal neocortical tissues from non-epileptic control subjects and patients with TLE. Specifically, we found that norbin colocalized with the dendritic marker microtubule-associated protein-2 (MAP2) (Fig. 1B). No glial fibrillary acidic protein (GFAP+) astrocytes were stained (data not shown), indicating that norbin was not expressed in these cells.

Regarding the western blotting results, norbin immunoreactivity bands were observed at approximately 75 kDa, and β-actin immunoreactivity bands were observed at approximately 42 kDa. We evaluated norbin expression in the temporal neocortex of all patients with TLE and all non-epileptic controls. The results confirmed that norbin is expressed at high levels in the temporal neocortex in patients without epilepsy and is expressed at low levels in this region in patients with TLE (Fig. 1C). The temporal neocortex samples from patients with TLE showed fainter norbin immunoreactivity than the normal brain tissue samples from non-epileptic autopsy control subjects (Fig. 1C). The mean optical density (OD) value for the norbin protein in the temporal neocortical tissues of patients with TLE was significantly lower than that in the corresponding tissues of non-epileptic autopsy control subjects (0.68 ± 0.21 vs. 1.29 ± 0.34, p < 0.01) (Fig. 1C).

### Norbin expression is decreased in the hippocampus and adjacent cortical tissues in epileptic rats

**Figure 2:**
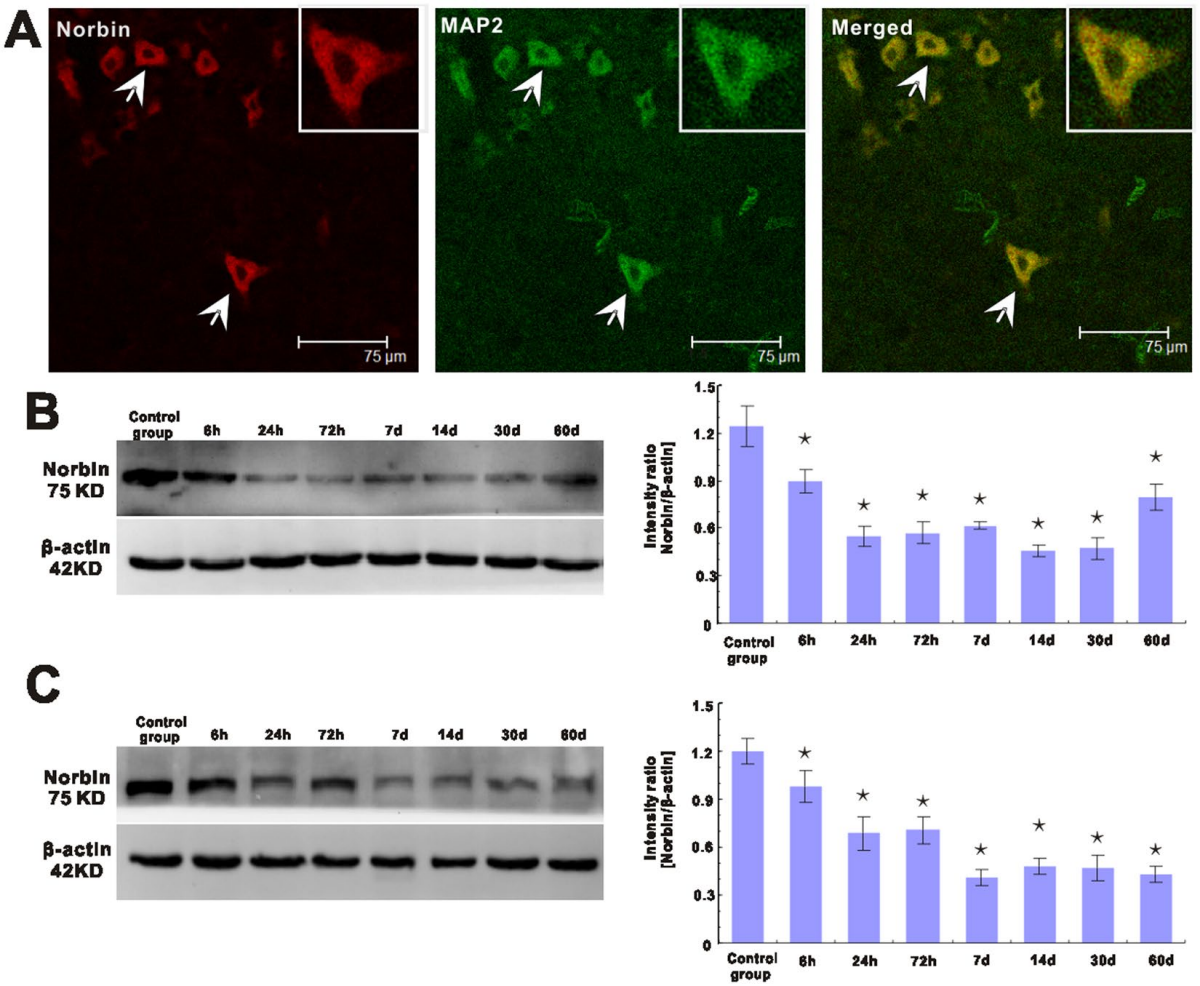
Double-immunofluorescence labeling and western blotting analysis of norbin expression in the hippocampus and adjacent cortical tissues of experimental rats (n = 56). (**A**) Norbin (red) and MAP2 (green) are coexpressed (merged) in the cortex of TLE rats. The arrowhead indicates a norbin+/MAP2+ cell. Insets show the representative morphology at a higher magnification. (**B**) Western blotting results for the hippocampal tissues of control rats (n = 7) and epileptic rats (n = 49). (Left) Representative western blotting images showing the bands corresponding to norbin (top) and β-actin (internal control, bottom) at different time points after SE. (Right) Comparison of the mean hippocampal immunoblotting OD value between control rats and epileptic rats at each time point after SE. *p < 0.05, epileptic rats at different time points vs. control. (**C**) Western blotting results for the cortical tissues of control (n = 7) and epileptic rats (n = 49). (Left) Representative western blotting images showing the bands corresponding to norbin (top) and β-actin (internal control, bottom) at different time points after SE. (Right) Comparison of the mean cortical immunoblotting OD value between control and epileptic rats at each time point after SE. *p < 0.05, epileptic rats at different time points vs. control.

To exclude the possibility that alterations in norbin expression may be caused by AEDs in patients with epilepsy, we performed experiments involving a rat model of epilepsy. We found that norbin was expressed exclusively in neurons of the hippocampus and adjacent cortical tissues in control and epileptic rats. Specifically, we noted that norbin colocalized with the dendritic marker MAP2 (Fig. 2A). GFAP+ astrocytes did not stain (data not shown), indicating that norbin was not expressed in these cells.

We performed western blot analysis to verify the above changes in norbin immunostaining. We found that decreases in norbin expression in the hippocampus and adjacent cortical tissues of epileptic rats began at 6 h post-status epilepticus (SE) onset and remained at a relatively low level until 60 days post-SE onset (Fig. 2B,C). Semiquantitative densitometric analysis revealed that norbin expression in the hippocampus and adjacent cortical tissues was significantly decreased at each time point in the corresponding group compared to the control group (p < 0.01) (Fig. 2B,C).

### p-CaMKII expression in epileptic brain

**Figure 3:**
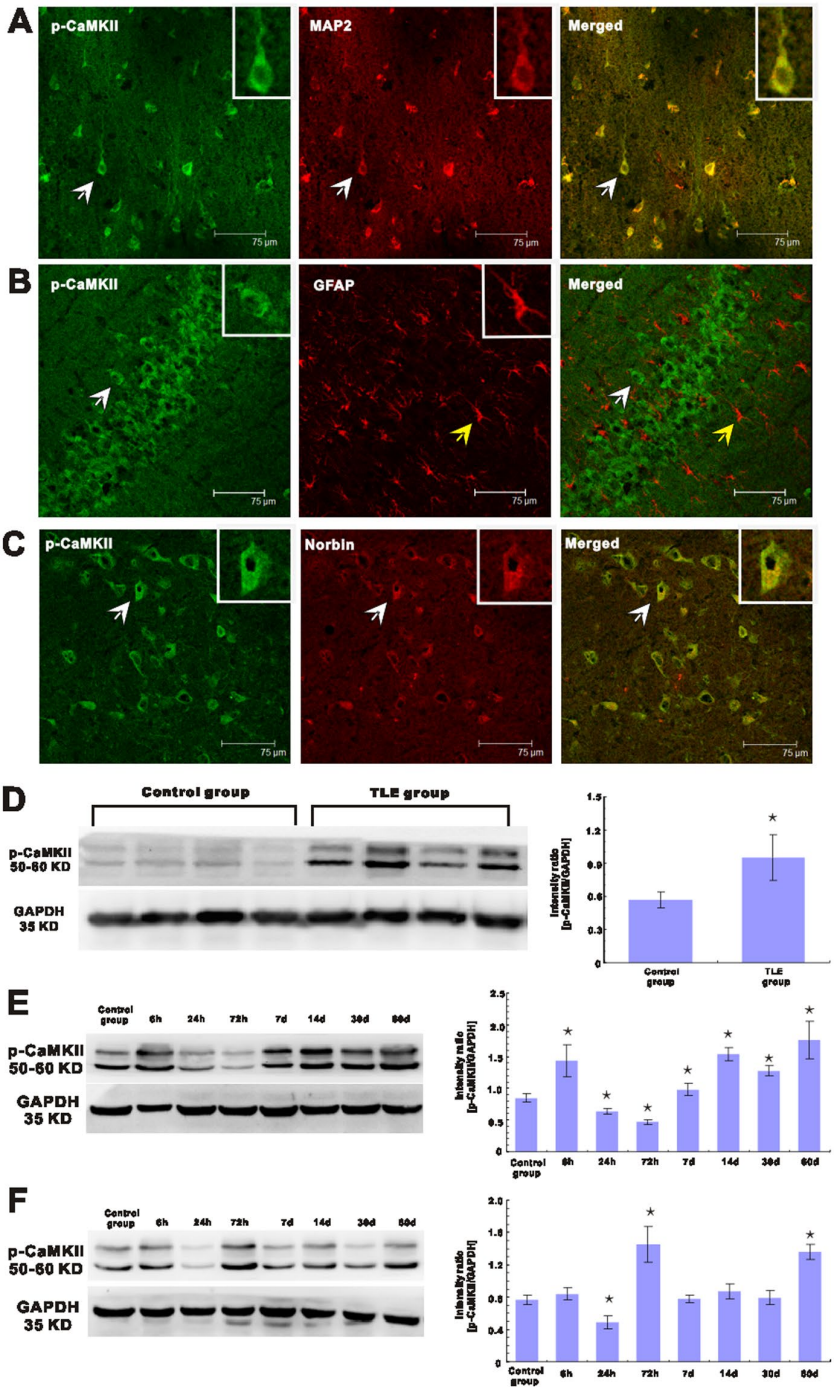
Double-immunofluorescence labeling and Western blotting analyses of p-CaMKII expression in patients and in the hippocampus and adjacent cortical tissues of experimental rats. (**A**) p-CaMKII (green) and MAP2 (red) are coexpressed (merged) in the temporal neocortex of patients with epilepsy. The arrowheads indicate a norbin+/MAP2+ cell. (**B**) p-CaMKII (green) and GFAP (red) are not coexpressed (merged) in the rat hippocampus. The white arrowhead indicates a p-CaMKII+ cell, and the yellow arrowhead indicates a GFAP+ cell. (**C**) p-CaMKII (green) and Norbin (red) are coexpressed (merged) in the cortex of TLE rats. (**D**) Western blotting analysis of p-CaMKII expression in the human temporal neocortex. (Left) Representative western blotting images showing the bands corresponding to p-CaMKII (top) and GAPDH (internal control, bottom) in patients with TLE and control subjects. (Right) Comparison of the mean OD value indicating that p-CaMKII is expressed at a significantly higher level in patients with TLE than in controls (*p < 0.05). (**E**) Western blotting results for the hippocampus of control and epileptic rats. (Left) Representative Western blotting images showing the bands corresponding to p-CaMKII (top) and GAPDH (bottom) at different time points after SE. (Right) Comparison of the mean hippocampal immunoblotting OD value between control rats and epileptic rats at each time point after SE. *p < 0.05, epileptic rats at different time points vs. control. (**F**) Western blotting results for the cortex of control rats and epileptic rats. (Left) Representative Western blotting images showing the bands corresponding to p-CaMKII (top) and GAPDH (bottom) at different time points after SE. (Right) Comparison of the mean cortical immunoblotting OD value between control rats and epileptic rats at each time point after SE. *p < 0.05, epileptic rats at different time points vs. control.

Using double-immunofluorescence labeling, we found that p-CaMKII was expressed exclusively in neurons in the temporal neocortical tissues of non-epileptic control subjects and patients with TLE. Specifically, we found that p-CaMKII colocalized with the dendritic marker MAP2 (Fig. 3A). No GFAP+ astrocytes stained (data not shown), indicating that p-CaMKII was not expressed in these cells.

We also found that p-CaMKII was expressed in neurons in epileptic rats. Specifically, we found that p-CaMKII colocalized with the dendritic marker MAP2 (data not shown). No GFAP+ astrocytes stained (Fig. 3B), indicating that p-CaMKII was not expressed in these cells.

Norbin is a negative regulator of p-CaMKII^4^, however, it is not clear the co-localization of p-CaMKII and norbin. We performed double staining immunofluorescence of both norbin and p-CaMKII to examine the localization of p-CaMKII and norbin in brain after epilepsy. Our studies showed that Norbin and p-CaMKII were co-expressed in single neuron (Fig. 3C).

Western blotting showed that p-CaMKII expression was significantly increased in patients with epilepsy compared to control subjects (Fig. 3D). p-CaMKII immunoreactive bands were observed at approximately 50–60 kDa, and GAPDH immunoreactive bands were observed at approximately 35 kDa. We evaluated p-CaMKII expression in the temporal neocortex of patients with TLE and non-epileptic controls. We found that the temporal neocortex samples from patients with TLE showed stronger p-CaMKII immunoreactivity than the normal brain tissue samples from non-epileptic autopsy controls (Fig. 3D). The difference in mean OD between the TLE and non-epileptic control groups was statistically significant (0.95 ± 0.21 vs. 0.56 ± 0.07, p < 0.01) (Fig. 3D).

Western blot analysis was performed to evaluate p-CaMKII expression in the hippocampus and cortex of epileptic rats. p-CaMKII expression in the indicated hippocampus homogenate increased at 6 h post-SE onset (169.6%, p < 0.01), decreased at 24 h and 72 h post-SE onset (p < 0.01) and then increased at 7 days post-SE onset and remaining elevated until 60 days post-SE onset (p < 0.01) (Fig. 3E). The differences in mean OD between the epileptic groups and non-epileptic control group were statistically significant (p < 0.01) (Fig. 3E). Western blot analysis was performed to evaluate p-CaMKII expression in the cortex of epileptic rats (Fig. 3F). p-CaMKII expression in the indicated epileptic tissues began to decrease at 24 h post-SE onset (p < 0.01) and then increased at 72 h post-SE onset (p < 0.01) and remained elevated until 60 days post-SE onset (p < 0.01) (Fig. 3F).

### Co-localization of norbin and mGluR5 in neurons of epileptic brain

**Figure 4:**
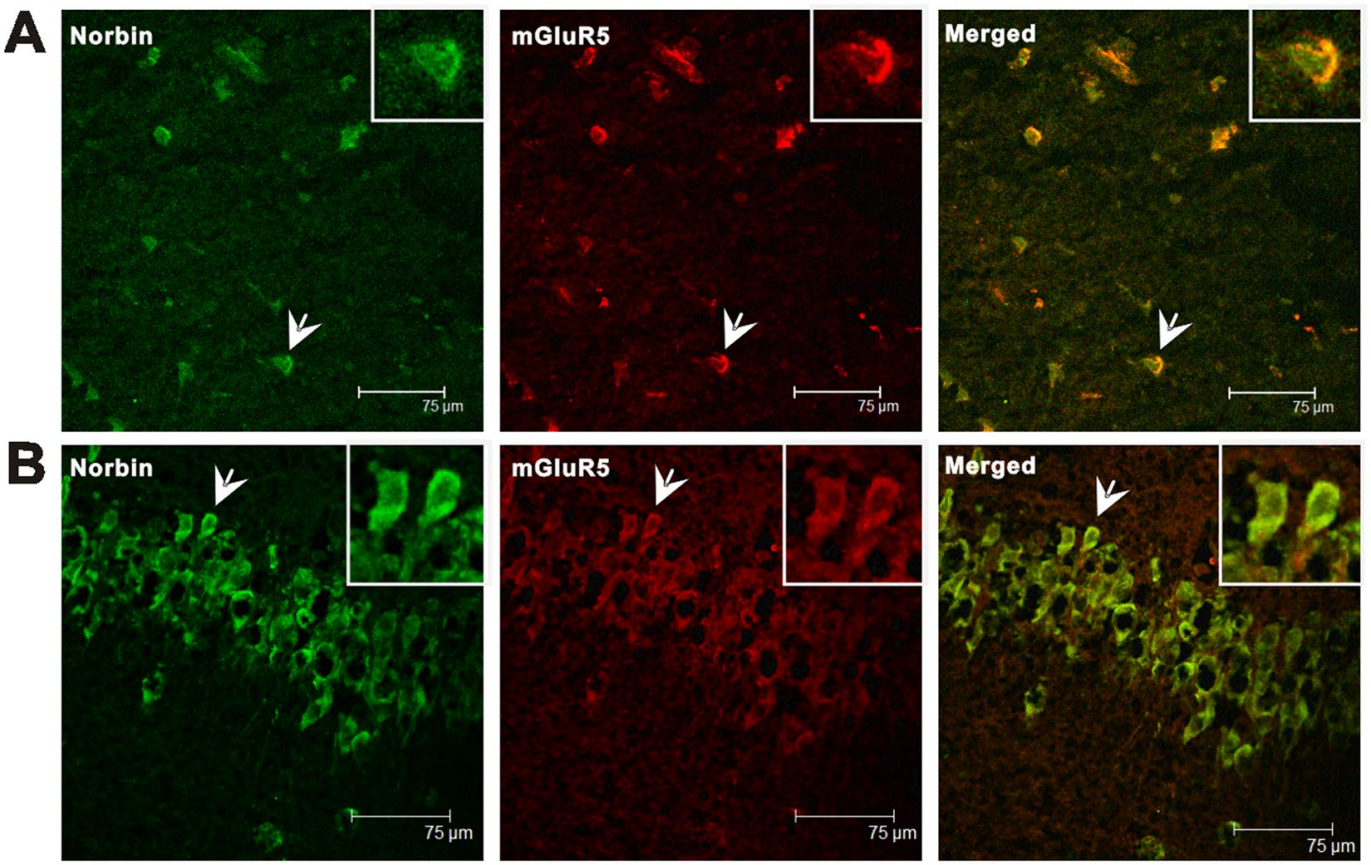
Double-immunofluorescence labelling for norbin and mGluR5 in the temporal neocortex of patients with TLE and in the hippocampus and adjacent cortical tissues of experimental rats. The arrowheads indicate norbin+/mGluR5+ cells. Insets show the representative morphology at a higher magnification. (**A**) Norbin (green) and mGluR5 (red) are coexpressed (merged) in the temporal neocortex of patients with epilepsy. (**B**) Norbin (green) and mGluR5 (red) are coexpressed (merged) in the hippocampus and adjacent cortical tissues of TLE rats.

Previous studies showed that norbin expression enhances mGluR5 signaling *in vitro*, norbin and mGluR5 are co-localization^3^. To examine localizations of mGluR5 and norbin in hippocampus and cortex after epilepsy, we used double staining immunofluorescence of mGluR5 and norbin in epileptic brain slice. Our studies found that mGluR5 and norbin were co-expressed in the neurons of temporal neocortical tissues from non-epileptic control subjects and patients with TLE. As shown in the indicated figure, mGluR5 colocalized with norbin (Fig. 4A). Our results also indicated that mGluR5 and norbin were co-expressed in the neurons of the rat hippocampus and adjacent cortical tissues (Fig. 4B).

### Adeno-associated viral (AAV) vectors in epilepsy

**Figure 5:**
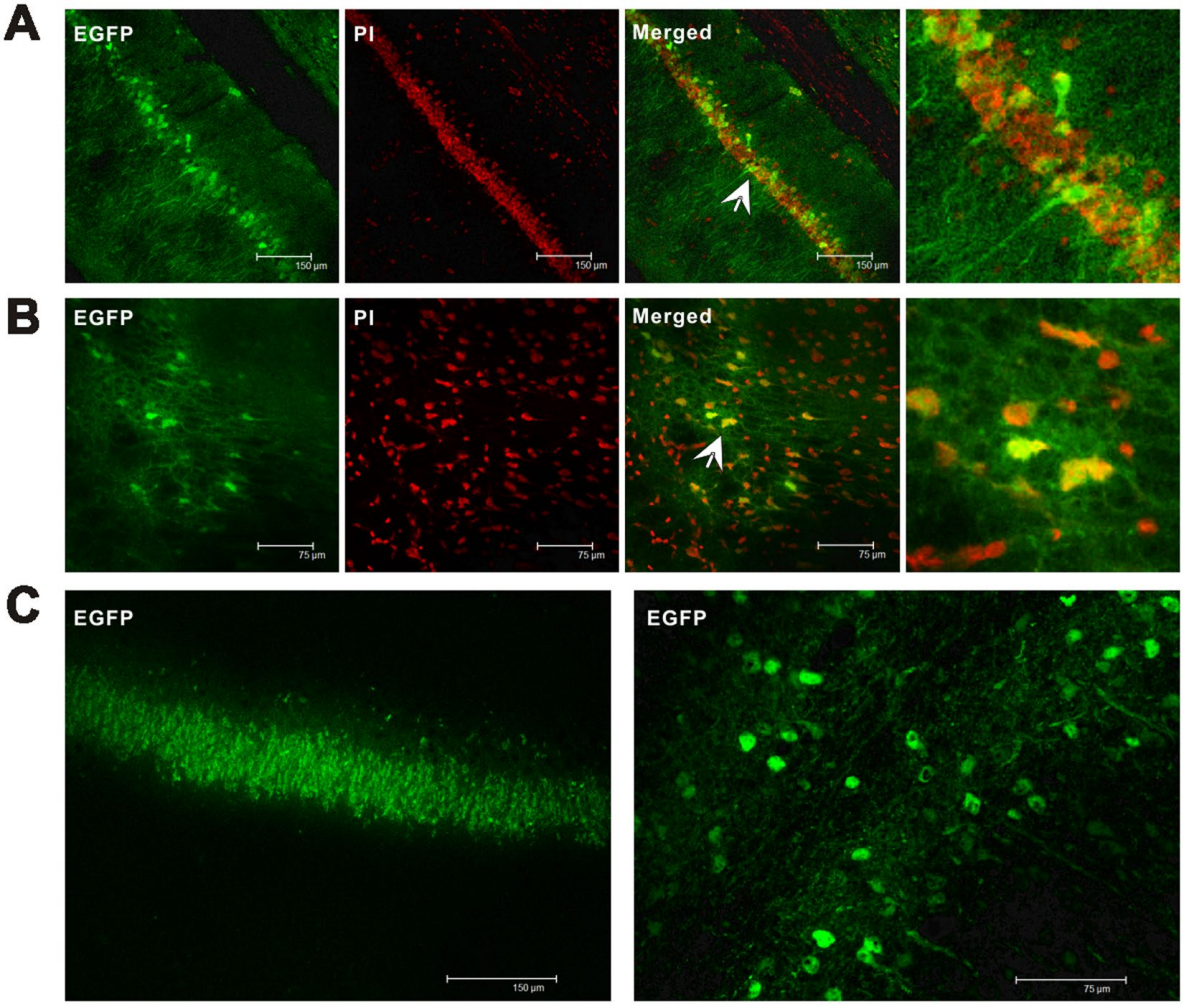
eGFP expression was assessed in the AAV-control brain slice by immunofluorescence and PI staining. (**A**) eGFP (green) and PI (red) are coexpressed (merged) in the hippocampus of rats. (**B**) eGFP (green) and PI (red) are coexpressed (merged) in cortex of rats. (**C**) eGFP expression was observed in the hippocampus and adjacent cortical tissues of rats after AAV-empty-vector injection.

**Figure 6:**
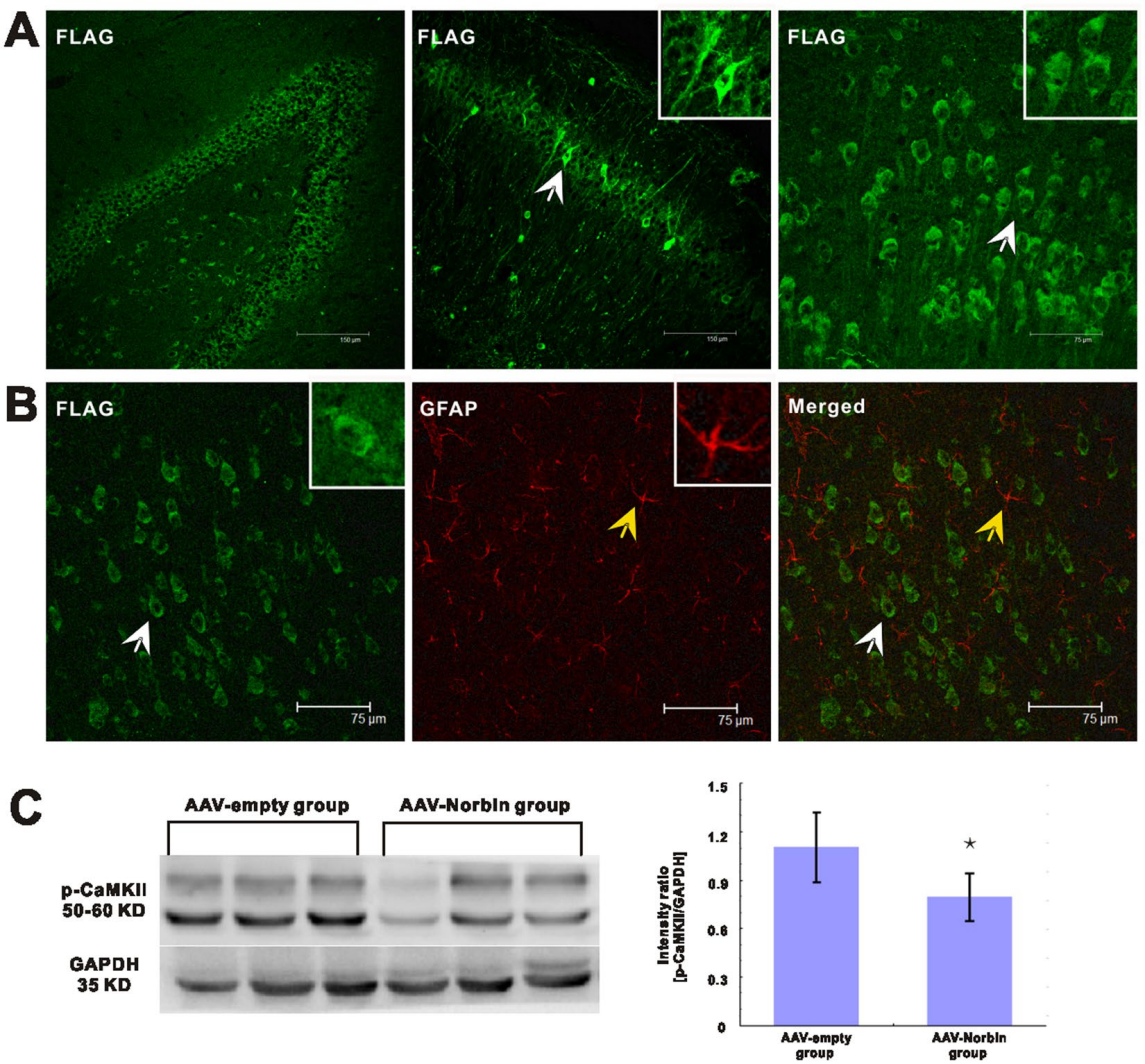
Immunofluorescence staining for Flag in the hippocampus and adjacent cortical tissues of experimental rats after AAV-Norbin-vector injection. Western blotting analyses of p-CaMKII expression in the hippocampus of rats after AAV-vectors injection. (**A**) Immunofluorescence staining for Flag in the rat hippocampus and adjacent cortical tissues after AAV-Norbin-vector injection. (**B**) Double immunofluorescence staining for Flag (green) and GFAP (red) in the rat cortex. Flag (green) and GFAP (red) are not coexpressed (merged) in the rat cortex. (**C**) Western blotting results for the hippocampus of rats after AAV-Norbin vector and AAV-empty vectors injection. (Left) Representative Western blotting images showing the bands corresponding to p-CaMKII (top) and GAPDH (bottom) at different group. (Right) Comparison of the mean hippocampal immunoblotting OD value between AAV-Norbin group and AAV-empty group (*p < 0.05, AAV-Norbin group vs. AAV-empty group).

At 3 days after vector injection, we observed enhanced green fluorescent protein (eGFP) expression via fluorescence microscopy. We found that eGFP was expressed in the hippocampus and adjacent cortical tissues and that its expression was stronger at 14 days after injection than at 3 days after injection (Fig. 5A,C). eGFP and the nuclear marker propidium iodide (PI) were expressed at the same location (Fig. 5A,B). Flag was expressed in the rat hippocampus and adjacent cortical tissues (Fig. 6A,B). Moreover, Flag was expressed exclusively in neurons and colocalized with the dendritic marker MAP2 (data not shown). No GFAP+ astrocytes were stained (Fig. 6B), indicating that Flag was not expressed in these cells.

We examined the expression of p-CaMKII in the hippocampus of rats at 14 days after injection. The expression of p-CaMKII was down regulation in the AAV-Norbin group than in the AAV-empty (control) group (Fig. 6C). Western blotting showed that p-CaMKII expression was significantly decreased in AAV-Norbin group compared to AAV-empty (control) group (Fig. 6C). We found that the hippocampus of rats after AAV-Norbin vector injection showed fainter p-CaMKII immunoreactivity than the hippocampus samples from rats after AAV-empty vector injection (Fig. 6C). The difference in mean OD between the AAV-Norbin and AAV-empty groups was statistically significant (1.10 ± 0.22 vs. 0.79 ± 0.19, p < 0.01) (Fig. 6C).

### Epileptic behavior

**Figure 7:**
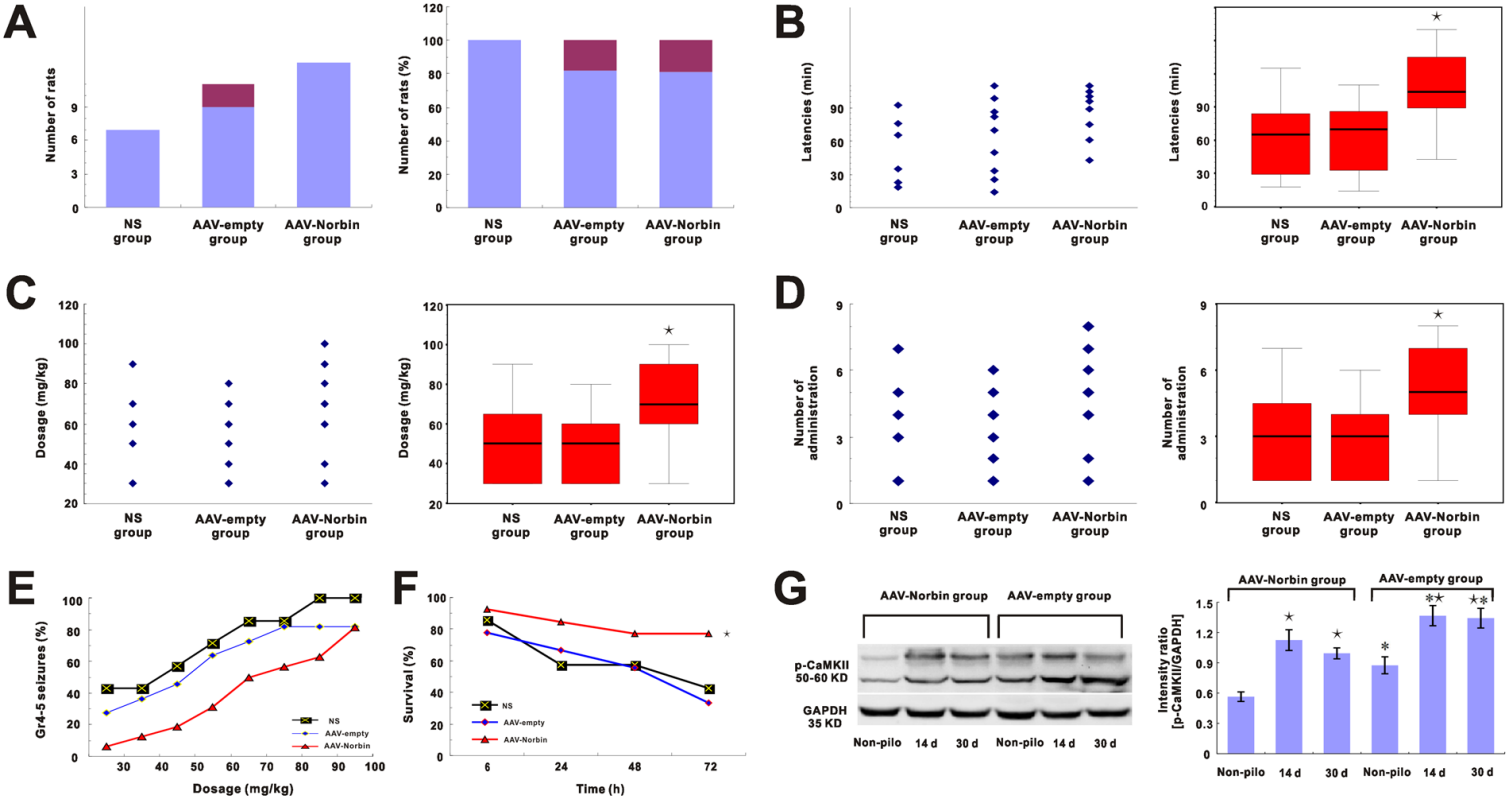
AAV-Norbin-vector-induced norbin over-expression in the rat hippocampus. (**A**) The number of rats that experienced grade 4–5 seizures in different groups. There were 7 out of 7 rats (NS group, n = 7), 9 out of 11 rats (AAV-empty group, n = 11), 13 out of 16 rats (AAV-norbin group, n = 16) show seizure grade 4–5. There was no difference in the number of rats that experienced grade 4–5 seizures (p > 0.05). (**B**) Comparison of latency among the three groups. Latency was longer in the AAV-norbin group than in the other groups (*p < 0.05) and was no difference between the NS and AAV-empty groups (p > 0.05). (**C**) The dosage of pilocarpine was required to induce grade 4–5 seizure. More pilocarpine was required to induce grade 4–5 seizure in the AAV-Norbin group than in the other groups (*p < 0.05). The dosage of pilocarpine required to induce grade 4–5 seizure was no difference between the NS and AAV-empty groups (p > 0.05). (**D**) The number of injection of pilocarpine required to induce grade 4–5 seizure. More pilocarpine injetions were required to induce grade 4–5 seizure in the AAV-Norbin group than in the other groups (*p < 0.05). The pilocarpine injections was no difference between the NS and AAV-empty groups (p > 0.05). (**E**) The percentage of rats that experienced grade 4–5 seizure in response to the same dose of pilocarpine, there was no significant difference among the three groups (p > 0.05). (**F**) Comparison of survival rates in the acute epileptic period. Survival was higher in the AAV-Norbin group than in the other groups (*p < 0.05). There was no difference in survival between the NS and AAV-empty groups (p > 0.05). (**G**) Western blotting for the hippocampus homogenates of non-pilocarpine treated rats and TLE rats at 14 day and 1 month after pilocarpine induced stages 4–5 seizures. (Left) Representative Western blotting images showing the bands corresponding to p-CaMKII (top) and GAPDH (bottom) at different time points after SE. (Right) Comparison of the mean immunoblotting OD value between non-pilocarpine treated rats and epileptic rats at each time point after SE in AAV-Norbin group and AAV-empty group. (*p < 0.05, AAV-Norbin group vs. AAV-empty group, *p < 0.05, non-pilocarpine treated rats vs epileptic rats).

In the AAV-Norbin group, 13 out of 16 (81.25%) rats displayed grade 4–5 seizure activity (81.25% of AAV-Norbin injected rats, n = 16). In the AAV-empty group, 9 out of 11 (81.82%) rats displayed grade 4–5 seizure activity (81.82% of AAV-empty injected rats, n = 11). In the saline group, 7 out of 7 (100%) rats displayed grade 4–5 seizure activity (n = 7). There was no significant difference in the percentage of rats that displayed grade 4–5 seizure activity among the three groups (Fig. 7A). Latency (min) was defined as the period between pilocarpine injection and the first grade 4–5 seizure. The AAV-Norbin group had a longer latency than the other groups (107.5 ± 35.7 vs. 62.0 ± 39.3, p < 0.05, 107 ± 35.7 vs. 63.1 ± 34.1, p < 0.05) (Fig. 7B). There was no difference in latency between the AAV-empty and NS groups. Comparison of the hydrochloride pilocarpine dose required to induce grade 4–5 seizures, the AAV-norbin group required more hydrochloride pilocarpine than the other groups (73.8 ± 22.6 vs. 51.4 ± 23.4, p < 0.05, 73.8 ± 22.6 vs.50.0 ± 18.7, p < 0.05) (Fig. 7C). There was no difference in the pilocarpine dose required to induce seizure activity between the AAV-empty and NS groups (p > 0.05).

More injections were required to induce seizures in the AAV-Norbin group than in the other two groups (5.4 ± 2.3 vs. 3.1 ± 2.3, p < 0.05, 5.4 ± 2.3 vs. 3.0 ± 2.3, p < 0.05) (Fig. 7D). There was no difference in the number of injections required to induce seizures between the AAV-empty and NS groups (p > 0.05).

We noted a significant difference among the three groups with respect to the percentage of rats that experienced grade 4–5 seizures after receiving the indicated dose of pilocarpine (30 mg/kg). However, we noted no significant difference among the three groups with respect to the percentage of rats that experienced grade 4–5 seizures in response to higher doses of pilocarpine (Fig. 7E).

Regarding survival among the rats that experienced grade 4–5 seizures within 72 h after pilocarpine administration, more rats in the AAV-Norbin group (10 out of 13 rats (76.92%) than in the other groups survived the acute epileptic period (p < 0.05). Three out of 7 rats (42.86%) in NS group and 3 out of 9 rats (33.33%) in AAV-empty group survived the acute epileptic period. There was no difference in survival between the AAV-empty and NS groups (p > 0.05) (Fig. 7F).

Our studies showed basal p-CaMKII was reduced in hippocampus of rats after AAV-Norbin overexpression (Fig. 6C). Furthermore, to show if AAV-Norbin overexpression blocks elevated p-CaMKII after pilocarpine hydrochloride induced seizures, we compared the p-CaMKII levels in the hippocampus homogenates of rats after pilocarpine hydrochloride induced seizures between AAV-Norbin group and AAV-empty group by Western bolt analysis. We analyzed the chronic periods of pilocarpine-induced epilepsy for mimics the epileptic state of patients. Our results showed that the p-CaMKII levels were increased at 14 day and at 1 month after pilocarpine-induced epilepsy both in AAV-Norbin group and AAV-empty group (Fig. 7G, p < 0.05). However, the p-CaMKII levels in AAV-Norbin group were lower than in AAV-empty group both in non-pilocarpine treated rats and in pilocarpine-induced epileptic rats (Fig. 7G, p < 0.05). The differences in mean OD between the AAV-Norbin and AAV-empty groups were statistically significant (14 d: 1.12 ± 0.12 vs. 1.37 ± 0.11, p < 0.05, 30 d: 0.99 ± 0.05 vs. 1.34 ± 0.10, p < 0.05) (Fig. 7G).

## Discussion

The major findings of this study were as follows: (i) norbin expression is significantly downregulated in the neurons of patients with TLE and lithium chloride-pilocarpine-treated rats, (ii) p-CaMKII expression is significantly upregulated in the neurons of patients with TLE and lithium chloride-pilocarpine-treated rats, (iii) norbin and mGluR5 were co-expressed in the neurons of patients with TLE and lithium chloride-pilocarpine-treated rats and (iv) norbin over-expression in the rat hippocampus results p-CaMKII down regulation, and influences epileptic behavior in lithium chloride-pilocarpine-treated rats.

Norbin modulates mGluR5 signaling^1^. mGluR5 responds mainly to the excitatory amino acid neurotransmitter glutamate, which localizes at presynaptic sites and plays important roles in normal brain function and several pathological disorders, including epilepsy. Although the mechanisms underlying epileptogenesis remain unknown, previous studies have shown that mGluR5 is involved in epileptogenesis^5^. Accumulating evidence indicate that that mGluR5 protein interactions play an important role in regulating mGluR5 trafficking, internalization and signaling^3^. mGluR5 availability is reduced in the brain after the onset of pilocarpine-induced SE^5,6^. In the present study, norbin expression was downregulated in patients and the animal model, and norbin and mGluR5 were co-expressed in the neurons of patients with TLE and lithium chloride-pilocarpine-treated rats. Epilepsy is caused by alterations in mGluR5-mediated glutamatergic neurotransmission. Decreases in the expression of norbin, which modulates mGluR5 signaling, may associated with the onset of epilepsy^1,3,7,8^. However, the exact role of norbin in the epilepsy phenotype remains unclear.

Norbin is a negative regulator of CaMKII (Thr-286) phosphorylation^4^. In our study, p-CaMKII expression was significantly up regulated in the neurons of patients with TLE. In lithium chloride-pilocarpine-treated rats, p-CaMKII expression decreased temporarily and then increased in chronic period. p-CaMKII was reduced in hippocampus of rats after AAV-Norbin vector injection. Over-expression of norbin results down regulation of p-CaMKII, which accord with norbin as a negative regulator p-CaMKII^4^. CaMKII is a multifunctional serine/threonine protein kinase in neurons that is involved in regulating neurotransmission and synaptic plasticity in response to neuronal activity-induced calcium signaling^9^. CaMKII-mediated modulation of neuronal sodium current has effect on neuronal excitability in epilepsy^10^, and its inactivation is associated with many experimental models of epilepsy^11,12^. CaKMII activity at synapses is associated with epileptogenesis, as well as other changes in the synapse that are related to glutamate receptor activation^13^. CaMKII is significantly upregulated in the hippocampi of patients with TLE^14^, pThr286-CaMKII was localized to the dentate granule cells bodies, which is similar immunohistochemistry labeling intensities in all TLE and control specimens^14^. However, other studies showed that expressions of CaMKII and p-CaMKII were not affected by convulsive status epilepticus in lithium chloride-pilocarpine-treated rats of 15-day-old and 35-day-old^15^, sodium valproate treatment *in vivo*, inhibited expression and phosphorylation of CaMKII^15^. CaMKII activity decreases in the immediate period (24 h) following seizure onset, a change that likely plays an important role in the development of recurrent seizure activity^13,16^. CaMKIIa mRNA expression also decreases following seizures; however, after CaMKII mRNA expression levels return to normal (36–48 h), corresponding increases in CaMKII protein expression levels also occur^13^. In this study, our results showed that norbin and p-CaMKII levels were negative correlation in hippocampus and cortex of control rats and epileptic rats at chronic periods (such as 1 month and 2 month), especially in hippocampus homogenate. However, the negative correlation is not well between norbin and p-CaMKII levels in hippocampus and cortex of epileptic rats at acute period. It is difficult to explain the phenomena, the possible reason maybe the protein changed later than DNA or mRNA changed in acute period or has other mechanisms.

Finally, we investigated the effects of norbin over-expression by injecting AAV vectors into the hippocampus of lithium chloride-pilocarpine model rats. Over-expression of norbin influences the expression of p-CaMKII, which reduced the expression of p-CaMKII in hippocampus of rats. We found that intra-hippocampal injections of AAV-norbin result in long-lasting protein over-expression in neurons, which influences seizure activity and protects rats during the acute epileptic period. Norbin over-expression also increased latency and survival in lithium chloride-pilocarpine model rats and prolonged latency in epilepsy, suggesting that norbin expression has antiepileptic effects.

We demonstrated for the first time that norbin is downregulated in the neurons of epileptic rat brains and patients with TLE. However, our study was not without limitations. First, only a select group of patients with TLE can be treated via surgical removal of their epileptogenic tissue. These pharmaco-resistant patients suffer from frequent seizures for many years and may not be representative of most patients with epilepsy. Second, we noted no significant difference in age between patients with TLE and control subjects; however, the TLE group included more adolescents than the control group. Structural and functional differences between adolescents and adults may contribute to the development of epilepsy in the former group. This phenomenon requires further study. Moreover, in our animal study, we used a lithium chloride-pilocarpine-induced epilepsy model. Pre-treatment of atropine will prevent chloride-pilocarpine-induced seizures^17^. The effects of norbin over-expression on epileptic behavior may be different in different epileptic models as a result of differences in the pathological changes induced by epilepsy in the models. Therefore, experiments with better designs are needed to study the effects of norbin over-expression on epilepsy in the future.

## Experimental Procedures

### Human brain tissue and clinical data

Our study protocol complied with the Guidelines for the Performance of Research Involving Human Subjects established by the National Institutes of Health and the Committee on Human Research at Chongqing Medical University. The study was approved by the Committee on Human Research of Chongqing Medical University.

Thirty-two patients undergoing surgery for medically intractable TLE and fourteen non-epilepsy control subjects were included in this study. All brain tissue specimens were randomly selected from our epilepsy brain tissue bank, which was described in our previous studies^18–20^. Written informed consent was obtained from all the patients or their legal guardians. All procedures were approved by the ethics committees of the appropriate institutions, and were conducted according to the guidelines of the Declaration of Helsinki.

Each patient underwent a presurgical assessment comprising a detailed history and neurological examination, interictal and ictal electroencephalographic studies, neuropsychological testing, and neuroradiological studies. At the time of surgery, all the patients with TLE were refractory to maximal doses of three or more AEDs, including Carbamazepine, Clonazepam, Lamotrigine, Phenobarbital, Phenytoin, Topiramate, and Valproate. All tissue blocks were obtained from patients who underwent therapeutic resections. After lesion resection, electrodes used for intraoperative electrocorticography were placed on the edges of the remaining tissue to ensure that the lesion had been completely resected.

Thirty-two human (fifteen females and seventeen males) brain samples were randomly selected from a bank of brain tissue samples taken from patients with intractable TLE whose ages ranged from 8 to 58 years. The mean age of the population was 24.4 ± 12.2 years. Table 1 summarizes the clinical features of these patients.

**Table 1:** Comparison of clinical data in the TLE patients and non-epileptic controls.

Clinical variable	TLE n = 32	Control n = 14	P-value
Age (years) (range)	24.4 ± 12.2 (8–58)	25.8 ± 11.5 (15–50)	0.609
Epilepsy onset (years) (range)	13.3 ± 9.7 (1–38)	NA	NA
Epilepsy duration (years) (range)	10.8 ± 7.4 (1–29)	NA	NA
Seizure frequency (no./month) (range)	9.9 ± 6.7 (3–30)	NA	NA
Seizure type (n)	CPS (9) SGS (17) CPS and SGS (6)	NA	NA
Male/female	17/15	8/6	0.529

CPS, complex partial seizures; SGS, secondary generalized seizures; Data are expressed as mean ± S.D. values. P-values were computed using independent samples t-test (age), or χ^2^ test (male/female). P-values < 0.05 were considered significant. Epilepsy duration was calculated as the time between epilepsy onset (onset of habitual seizures) and surgery. NA = not applicable.

For comparison purposes, we obtained fourteen histologically normal temporal neocortex samples from individuals who were treated for increased intracranial pressure caused by head trauma from a traffic accident and later died from their injuries. Control brain samples were collected only in cases in which the brain was acutely exposed to the environment outside the skull as a result of a severe head injury, the patient was deemed unrevivable, or the patient had died. Conventional neuropathologic examinations revealed no signs of central nervous system disease in the brain tissues of the control group, whose mean age was 25.8 ± 11.5 years (range, 15–50 years). The control subjects had no history of epilepsy or exposure to AEDs. There were no significant differences in age, sex, or tissue topography between the TLE and control tissue samples.

### Rat model of epilepsy

All animal procedures were conducted in accordance with international standards and were approved by the Commission for Ethical Experimentation on Animals of Chongqing Medical University.

The rat model was established as reported previously^19,20^. Healthy adult male Sprague–Dawley rats (n = 56; obtained from the Chongqing Medical University Laboratory Animal Center) weighing 250–300 g were randomly assigned to a normal control group (n = 7) or an experimental group (n = 49). Approximately 18 h thereafter, we treated the animals with atropine sulfate (1 mg/kg, i.p.) to limit the peripheral effects of the convulsant. Thirty min later, we induced status epilepticus (SE) in 49 rats by injecting them with pilocarpine hydrochloride (30 mg/kg, i.p., Sigma, USA). Pilocarpine hydrochloride was administered (10 mg/kg, i.p.) every 30 min until the rats developed seizures. The rats developed spontaneous recurrent seizures, which were scored according to Racine, at 6–11 days after pilocarpine treatment^21^. Only rats that attained grade 4–5 seizures were included in the study. Seven control rats were treated with lithium chloride and atropine sulfate but were subsequently treated with saline instead of pilocarpine. One hour after the onset of SE, we reduced the severity of the convulsions from which the rats were suffering with 10 mg/kg diazepam i.p. The experimental group was randomly divided into 6-h, 24-h, 72-h, 7-day, 14-day, 30-day, and 60-day post-SE onset subgroups; thus, the rats in these groups were sacrificed at 6 h, 24 h, 72 h, 7 days, 14 days, 30 days, or 60 days after SE onset, after which the hippocampus and adjacent cortex were removed for study.

### Tissue processing

In both the human and animal experiments, one portion of resected brain tissue was immediately fixed in 10% buffered formalin for 48 h. The tissue specimens were then embedded in paraffin and sectioned at a thickness of 5 μm for the immunohistochemistry experiments and a thickness of 10 μm for the double-immunofluorescence labeling analysis. The other portions of resected brain tissue were immediately stored in liquid nitrogen and later used for protein extraction (see the western blotting protocol). The animals were subsequently perfused transcardially with physiological saline, followed by 4% paraformaldehyde in 0.1 M phosphate buffer (PB, pH 7.4), under chloral hydrate anesthesia (0.35 g/kg, i.p.). The brains were removed and postfixed in the same fixative for 1 h. Thereafter, the entire hippocampus and adjacent cortex were frozen and sectioned at a thickness of 10 μm with a cryostat for double-immunofluorescence labeling analysis.

### Double-immunofluorescence labeling

The tissue sections were deparaffinized, rehydrated in a graded ethanol series, and then incubated in H_2_O_2_ (0.3%, 15 min). The frozen sections were subsequently air-dried on a slide warmer at 50 °C for at least half an hour. For antigen retrieval, the sections were treated with 10 mmol/l sodium citrate buffer (pH 6.0) and heated in a microwave oven for 20 min at 92–98 °C. The tissues were then permeabilized with 0.5% Triton X-100, after which they were incubated in serum for 1 h at room temperature. The sections were then incubated with a mixture of primary antibodies overnight at 4 °C.

The sections were subsequently incubated with the following antibodies: anti-norbin antibody (Abcam, USA), rabbit anti-MAP2 antibody (Santa Cruz, USA), polyclonal goat anti-norbin antibody (Abcam, USA), rabbit anti-GFAP antibody (Santa Cruz, USA), polyclonal goat anti-norbin antibody (Santa Cruz, USA), rabbit anti-mGluR5 antibody (Santa Cruz, USA), rabbit anti-p-CaMKII antibody (phosphor T286) (Abcam, USA), mouse anti-GFAP antibody (Santa Cruz, USA), rabbit anti-p-CaMKII antibody (phosphor T286) (Abcam, USA), mouse anti-MAP2 antibody (Santa Cruz, USA), mouse anti-Flag antibody, rabbit anti-MAP2 antibody (Santa Cruz, USA), mouse anti-Flag antibody and rabbit anti-GFAP antibody (Santa Cruz, USA). The sections were then washed and incubated with the appropriate secondary antibody, i.e., fluorescein isothiocyanate (FITC)-conjugated IgG (Zhongshan Golden Bridge Inc. Beijing, China) or tetramethylrhodamine isothiocyanate (TRITC)-conjugated IgG (Zhongshan Golden Bridge Inc. Beijing, China), in the dark for 60 min at room temperature. For propidium iodide (PI) staining, the slides were incubated in 2% PI in the dark for 7 min. After being rinsed thrice for 1 min each in distilled water, the slides were dried and coverslipped with 50% glycerol/PBS. Fluorescence was detected by laser scanning confocal microscopy (Leica Microsystems Heidelberg GmbH, Germany), which was performed with an Olympus IX70 inverted microscope (Olympus) equipped with a Fluoview FVX confocal scanhead.

### Immunohistochemistry

Immunohistochemical staining was conducted using the avidin-biotin-peroxidase complex method, according to established protocols or the manufacturer’s instructions. The primary antibody used in the experiment was mouse anti-norbin, and the secondary antibody used in the experiment was included with the indicated kit (Wuhan Boster Biological Technology, Wuhan, China). The negative controls were incubated with PBS rather than the indicated primary antibody. The images of each section were scanned and acquired by an OLYMPUS PM20 automatic microscope (Olympus, Japan) and a TCFY-2050 (Yuancheng Inc., China) pathology system. Five random visual fields in each section were imaged (5 sections in each brain). The immunohistochemical results were assessed by automatically measuring the average integrated optical density (IOD) to determine the IOD-to-field area ratio using a Motic Med 6.0 CMIAS pathology image analysis system (Beihang Motic Inc., China)^19^. The mean of the values from 5 fields in each slide image was subsequently calculated and used to determine the differences in the immunohistochemistry results between the epilepsy and control groups.

### Western blotting

Fifty micrograms of protein were separated by 10% SDS–PAGE and then transferred to polyvinylidene fluoride (PVDF) membranes (Millipore Corporation, USA) for western blot analysis, which was performed with a Bio-Rad apparatus (Bio-Rad, USA). Non-specific epitopes were blocked with 5% skim milk/Tween-20-Tris-buffered saline (TTBS), after which the membranes were incubated with each of the following primary antibodies overnight at 4 °C: mouse anti-norbin antibody (1:500, Abcam, USA), rabbit anti-p-CaMKII antibody (1:1000), rabbit anti-β-actin as control antibody (1:5000, Beijing 4A Biotech Co., Ltd, Beijing, China) and mouse anti-GAPDH antibody (1:4000). After being washed thrice in TBST, the membranes were treated with HRP-conjugated secondary antibodies for 1 h at room temperature before being visualized in a darkroom with an ECM kit (Pierce, USA) and a CCD camera (Bio-Rad Laboratories, USA). The digitally scanned immunoblots were subsequently analyzed using Quantity One software (Bio-Rad Laboratories, USA). The band intensities for norbin and p-CaMKII, which were represented by the ODs of norbin and p-CaMKII blot expression, were normalized to those of β-actin or GAPDH after electrophoresis^22^.

### Norbin over-expression by AAV vector injection

The AAV vectors used herein were derived from a mixture of adenoviral serotypes 2 and 8. One of the vectors included the full-length cDNA sequence encoding rat norbin (stock solutions: AAV- Norbin, 1.26E + 13 genomic particles/ml), and the other vector served as a control (AAV-empty: 6.72E + 13 genomic particles/ml). The AAV-Norbin and AAV-empty vectors were generated, produced and purified by Neuron Biotech Co., Ltd. (Shanghai, China). Briefly, the target gene was cloned into an AAFE001BpAOV.Syn. eGFP AAV production shuttle plasmid, with which AAV-293 cells were subsequently infected to produce AAV vectors, which were purified by double-CsCl ultracentrifugation and then buffer-exchanged with PD-10 desalting columns. The AAV titers were determined by quantitative real-time PCR, and the purity of the nucleic acid sequences contained therein was verified by sodium dodecyl sulfate–polyacrylamide gel electrophoresis. Supplement 1 shows an AAV vector spectrum graph (supplement 1), and supplement 2 (supplement 2) shows the AAV vector sequence. Studies have shown that AAV-empty has no effect on seizure activity, nor does it have an effect on related neuropathology; thus, its effects, or lack thereof, were similar to those of saline or vectors carrying reporter genes (such as GFP)^23–25^.

The rats were anesthetized by intraperitoneal injections of chloral hydrate (350 mg/kg, Sigma-Aldrich, St Louis, MO, USA) and then placed in a stereotaxic frame (Woruide, Shenzhen, Guangdong, China), after which the AAV-vectors were injected using a 30-gauge needle. Two microliters of viral vector suspension and 1 µl of a mannitol mixture were infused into the dorsal (anterior–posterior, −3.3 mm; medial–lateral, 1.8 mm; dorsal–ventral, −2.6 mm) and ventral hippocampi bilaterally (anterior–posterior, −4.8 mm; medial–lateral, 5.2 mm; dorsal–ventral, −6.4 mm; and −3.8 mm; 0.75 µl at each location in the dorsal–ventral plane)^23,26,27^. The bregma was the reference point for the anterior–posterior axis, the midline was reference point for the medial–lateral axis, and the dura was the reference point for the dorsal–ventral axis. The pipette was left in place for an additional 5 min after the injection to prevent backflow of the viral particles through the injection track^26^. The viral vector stock solutions were diluted 1:10 with sterile phosphate-buffered saline before use.

After 2–3 weeks, some of rats were sacrificed via the infusion of drugs into the left ventricle. eGFP expression was subsequently assessed in the AAV-control brain slice, and Flag expression was assessed in the AAV-Norbin brain slice by immunofluorescence. The other portions of resected brain tissue were immediately stored in liquid nitrogen and later used for protein extraction (see the western blotting protocol). At the same time point, the epileptic rat model was induced by lithium chloride-pilocarpine hydrochloride administration, after which kindling was performed, according to a previously described protocol. The initial pilocarpine hydrochloride dose was 30 mg/kg. At 45 minutes after the first dose, pilocarpine hydrochloride was administered at the indicated dose (10 mg/kg, i.p.), which was re-administered every 15 minutes thereafter until the rats developed seizures. Rats that did not experience grade 4–5 seizures after 8 pilocarpine injections were classified as non-seizure rats. After 2 h of SE, the seizure rats were given 10 mg/kg diazepam i.p. to stop their seizures. The animals were then observed at least for 72 h, after which their brains were quickly removed and processed as described previously.

### Statistical analysis

Data were expressed as the mean ± standard deviation (S.D.), and the analysis of the differences between the TLE and control groups was conducted using independent samples t-tests (age) or χ2 tests (male/female) (SPSS 11.5). The significance of the differences among more than 2 groups was determined by one-way ANOVA followed by Bonferroni’s post hoc test for multiple comparisons. p < 0.05 was considered statistically significant.

## Electronic supplementary material

Supplementary information
