# Supplementary material for: Altered Norbin Expression in Patients with Epilepsy and a Rat Model
Source: Sci Rep. 2017 Oct 25;7:13970. doi: 10.1038/s41598-017-13248-9 (PMC5656659; doi:10.1038/s41598-017-13248-9)
Supplement: Supplementary file 1 — Supplementary information [file 41598_2017_13248_MOESM1_ESM.pdf]

Altered Norbin Expression in Patients with Epilepsy and a Rat Model

Yali Xu[1,2], Zengyou Li[2], Li Yao[3], Xingping Zhang[1], Dan Gan[1], Manchun Jiang[1], Na Wang[2], Guojun Chen[2], Xuefeng Wang[2*]

1. Department of Gerontology, Chongqing General Hospital, 104 Loquat Mountain Street, Chongqing, China

2. Department of Neurology, The First Affiliated Hospital, Chongqing Medical University, 1 Youyi Road, Chongqing, China

3. Health Checkup Center, Chongqing General Hospital, 104 Loquat Mountain Street, Chongqing, China

Yali Xu and Zengyou Li contributed equally to this work

corresponding author: Xuefeng Wang[2*]

E-mail address: xfyp@163.com (X.-F. Wang)

Tel & Fax: +86 23 6870 8697

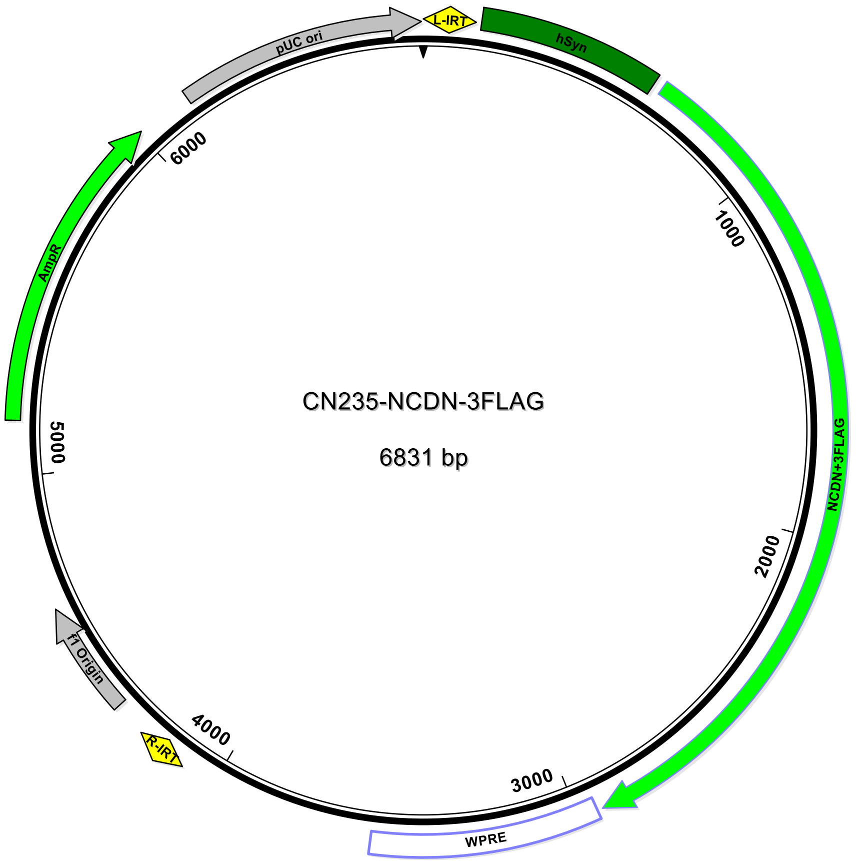

Supplement Figure 1: Spectrum of a graph of AAV vector (Rat NCDN Expression AAV2/8)

```
                                                     10        20        30        40        50        60
                                                AAAGATGCCAACTCCATCACTAGGGGTCCTGCGGCCGCACGCGTGTGTCTAGACTGCAGAGGGCC
CN235_1_(0708-107)235-4_E11_11(16>1030)    ←    AAAGATGCCAACTCCATCACTAGGGGTCCTGCGGCCGCACGCGTGTGTCTAGACTGCAGAGGGCC

                                                70        80        90       100       110       120       130
                                                TGCGTATGAGTGCAAGTGGGTTTTAGGACCAGGATGAGGCGGGGTGGGGGTGCCTACCTGACGAC
CN235_1_(0708-107)235-4_E11_11(16>1030)    ←    TGCGTATGAGTGCAAGTGGGTTTTAGGACCAGGATGAGGCGGGGTGGGGGTGCCTACCTGACGAC

                                                     140       150       160       170       180       190
                                                CGACCCCGACCCACTGGACAAGCACCCAACCCCCATTCCCCAAATTGCGCATCCCCTATCAGAGA
CN235_1_(0708-107)235-4_E11_11(16>1030)    ←    CGACCCCGACCCACTGGACAAGCACCCAACCCCCATTCCCCAAATTGCGCATCCCCTATCAGAGA

                                                200       210       220       230       240       250       260
                                                GGGGGAGGGGAAACAGGATGCGGCGAGGCGCGTGCGCACTGCCAGCTTCAGCACCGCGGACAGTG
CN235_1_(0708-107)235-4_E11_11(16>1030)    ←    GGGGGAGGGGAAACAGGATGCGGCGAGGCGCGTGCGCACTGCCAGCTTCAGCACCGCGGACAGTG

                                                     270       280       290       300       310       320
                                                CCTTCGCCCCCGCCTGGCGGCGCGCGCCACCGCCGCCTCAGCACTGAAGGCGCGCTGACGTCACT
CN235_1_(0708-107)235-4_E11_11(16>1030)    ←    CCTTCGCCCCCGCCTGGCGGCGCGCGCCACCGCCGCCTCAGCACTGAAGGCGCGCTGACGTCACT

                                                330       340       350       360       370       380       390
                                                CGCCGGTCCCCCGCAAACTCCCCTTCCCGGCCACCTTGGTCGCGTCCGCGCCGCCGCCGGCCCAG
CN235_1_(0708-107)235-4_E11_11(16>1030)    ←    CGCCGGTCCCCCGCAAACTCCCCTTCCCGGCCACCTTGGTCGCGTCCGCGCCGCCGCCGGCCCAG

                                                     400       410       420       430       440       450
                                                CCGGACCGCACCACGCGAGGCGCGAGATAGGGGGGCACGGGCGCGACCATCTGCGCTGCGGCGCC
CN235_1_(0708-107)235-4_E11_11(16>1030)    ←    CCGGACCGCACCACGCGAGGCGCGAGATAGGGGGGCACGGGCGCGACCATCTGCGCTGCGGCGCC

                                                460       470       480       490       500       510       520
                                                GGCGACTCAGCGCTGCCTCAGTCTGCGGTGGGCAGCGGAGGAGTCGTGTCGTGCCTGAGAGCGCA
CN235_1_(0708-107)235-4_E11_11(16>1030)    ←    GGCGACTCAGCGCTGCCTCAGTCTGCGGTGGGCAGCGGAGGAGTCGTGTCGTGCCTGAGAGCGCA

                                                     530       540       550       560       570       580
                                                GTCGAGAAGGTACCGGATCCCCGGGTACCGGTCGCCACCATGTCGTGTTGTGACCTGGCTGCGGC
CN235_1_(0708-107)235-4_E11_11(16>1030)    ←    GTCGAGAAGGTACCGGATCCCCGGGTACCGGTCGCCACCATGTCGTGTTGTGACCTGGCTGCGGC
CN235.seq(1>2269)                          →                                    cgccaccatgtcgtgttgtgacctggctgcggc

                                                590       600       610       620       630       640       650
                                                GGGACAGTTGGGCAAGGCGGGCATCATGGCCTCGGATTGTGAGCCAGCTCTGAACCAGGCAGAGA
CN235_1_(0708-107)235-4_E11_11(16>1030)    ←    GGGACAGTTGGGCAAGGCGGGCATCATGGCCTCGGATTGTGAGCCAGCTCTGAACCAGGCAGAGA
CN235.seq(1>2269)                          →    gggacagttgggcaaggcgggcatcatggcctcggattgtgagccagctctgaaccaggcagaga

                                                     660       670       680       690       700       710
                                                GCCGAAACCCCACCCTGGAGCGCTACCTGGGAGCCCTCCGTGAGGCCAAGAATGACAGCGAGCAG
CN235_1_(0708-107)235-4_E11_11(16>1030)    ←    GCCGAAACCCCACCCTGGAGCGCTACCTGGGAGCCCTCCGTGAGGCCAAGAATGACAGCGAGCAG
CN235.seq(1>2269)                          →    gccgaaaccccaccctggagcgctacctgggagccctccgtgaggccaagaatgacagcgagcag

                                                720       730       740       750       760       770       780
                                                TTTGCAGCCCTGCTGCTAGTAACCAAGGCAGTCAAAGCAGGTGACATTGACGCCAAAACTCGACG
CN235_1_(0708-107)235-4_E11_11(16>1030)    ←    TTTGCAGCCCTGCTGCTAGTAACCAAGGCAGTCAAAGCAGGTGACATTGACGCCAAAACTCGACG
CN235.seq(1>2269)                          →    tttgcagccctgctgctagtaaccaaggcagtcaaagcaggtgacattgacgccaaaactcgacg

                                                     790       800       810       820       830       840
                                                TAGGATCTTTGATGCTGTTGGGTTCACCTTTCCCAACCGACTCCTGACTACTAAGGAGGCCCCTG
CN235_1_(0708-107)235-4_E11_11(16>1030)    ←    TAGGATCTTTGATGCTGTTGGGTTCACCTTTCCCAACCGACTCCTGACTACTAAGGAGGCCCCTG
CN235.seq(1>2269)                          →    taggatctttgatgctgttgggttcacctttcccaaccgactcctgactactaaggaggcccctg

                                                850       860       870       880       890       900       910
                                                ATGGCTGCCCTGACCACGTTCTCCGGGCCCTGGGCGTGGCCCTGCTGGCCTGTTTCTGCAGCGAC
CN235_1_(0708-107)235-4_E11_11(16>1030)    ←    ATGGCTGCCCTGACCACGTTCTCCGGGCCCTGGGCGTGGCCCTGCTGGCCTGTTTCTGCAGCGAC
CN235.seq(1>2269)                          →    atggctgccctgaccacgttctccgggccctgggcgtggccctgctggcctgtttctgcagcgac
```

```
                                                    920       930       940       950       960       970
                                                CCTGAACTAGCCAGCCATCCCCAGGTCCTGAACAAGATCCCCATCCTTTGCACATTCCTGACAGC
CN235_1_(0708-107)235-4_E11_11(16>1030)   ←     CCTGAACTAGCCAGCCATCCCCAGGTCCTGAACAAGATCCCCATCCTTTGCACATTCCTGACAGC
CN235.seq(1>2269)                         →     cctgaactagccagccatccccaggtcctgaacaagatccccatcctttgcacattcctgacagc

                                                980       990       1000      1010      1020      1030      1040
                                                CCGAGGGGATCCTGATGATGCTGCCCGCCGCTCCATgATtGATGACaCCTACCAGTGCCTGACAG
CN235_1_(0708-107)235-4_E11_11(16>1030)   ←     CCGAGGGGATCCTGATGATGCTGCCCGCCGCTCCATGAT-G
CN235.seq(1>2269)                         →     ccgaggggatcctgatgatgctgcccgccgctccatgattgatgacacctaccagtgcctgacag
CN235_1_(0708-108)NCDN-1_D11_11(7>925)    →                                         T-AATGATGAC-CCTACCAGTGCCTGACAG

                                                    1050      1060      1070      1080      1090      1100
                                                CTGTTGCAGGCACACCCCGAGGGCCCCGACACCTCATTGCTGGTGGCACAGTGTCTGCCCTGTGC
CN235.seq(1>2269)                         →     ctgttgcaggcacaccccgagggccccgacacctcattgctggtggcacagtgtctgccctgtgc
CN235_1_(0708-108)NCDN-1_D11_11(7>925)    →     CTGTTGCAGGCACACCCCGAGGGCCCCGACACCTCATTGCTGGTGGCACAGTGTCTGCCCTGTGC

                                                1110      1120      1130      1140      1150      1160      1170
                                                CAGGCATACCTGGGGCATGGCTATGGCTTTGACCAGGCCCTGGCACTCCTGGTGGGGCTGCTGGC
CN235.seq(1>2269)                         →     caggcatacctggggcatggctatggctttgaccaggccctggcactcctggtggggctgctggc
CN235_1_(0708-108)NCDN-1_D11_11(7>925)    →     CAGGCATACCTGGGGCATGGCTATGGCTTTGACCAGGCCCTGGCACTCCTGGTGGGGCTGCTGGC

                                                    1180      1190      1200      1210      1220      1230
                                                TGCTGCAGAGACACAGTGCTGGAAGGAGGCAGAGCCCGACCTGCTGGCTGTGTTGCGAGGCCTCA
CN235.seq(1>2269)                         →     tgctgcagagacacagtgctggaaggaggcagagcccgacctgctggctgtgttgcgaggcctca
CN235_1_(0708-108)NCDN-1_D11_11(7>925)    →     TGCTGCAGAGACACAGTGCTGGAAGGAGGCAGAGCCCGACCTGCTGGCTGTGTTGCGAGGCCTCA

                                                1240      1250      1260      1270      1280      1290      1300
                                                GCGAGGATTTCCAAAGAGCTGAAGATGCCAGCAAGTTTGAGCTCTGCCAGCTGCTGCCCCTTTTC
CN235.seq(1>2269)                         →     gcgaggatttccaaagagctgaagatgccagcaagtttgagctctgccagctgctgccccttttc
CN235_1_(0708-108)NCDN-1_D11_11(7>925)    →     GCGAGGATTTCCAAAGAGCTGAAGATGCCAGCAAGTTTGAGCTCTGCCAGCTGCTGCCCCTTTTC

                                                    1310      1320      1330      1340      1350      1360
                                                CTGCCCCCAACAACTGTGCCCCCTGAATGCCACCGGGATCTGCAGGCTGGGCTGGCACGCATCCT
CN235.seq(1>2269)                         →     ctgcccccaacaactgtgccccctgaatgccaccgggatctgcaggctgggctggcacgcatcct
CN235_1_(0708-108)NCDN-1_D11_11(7>925)    →     CTGCCCCCAACAACTGTGCCCCCTGAATGCCACCGGGATCTGCAGGCTGGGCTGGCACGCATCCT

                                                1370      1380      1390      1400      1410      1420      1430
                                                AGGAAGCAAGTTGAGCTCCTGGCAGCGCAATCCTGCACTGAAGCTGGCAGCCCGCCTGGCTCATG
CN235.seq(1>2269)                         →     aggaagcaagttgagctcctggcagcgcaatcctgcactgaagctggcagcccgcctggctcatg
CN235_1_(0708-108)NCDN-1_D11_11(7>925)    →     AGGAAGCAAGTTGAGCTCCTGGCAGCGCAATCCTGCACTGAAGCTGGCAGCCCGCCTGGCTCATG

                                                    1440      1450      1460      1470      1480      1490
                                                CCTGCGGCTCCGACTGGATCCCAGTGGGCAGCTCTGGGAGCAAGTTTCTGGCCCTCCTGGTGAAT
CN235.seq(1>2269)                         →     cctgcggctccgactggatcccagtgggcagctctgggagcaagtttctggccctcctggtgaat
CN235_1_(0708-108)NCDN-1_D11_11(7>925)    →     CCTGCGGCTCCGACTGGATCCCAGTGGGCAGCTCTGGGAGCAAGTTTCTGGCCCTCCTGGTGAAT

                                                1500      1510      1520      1530      1540      1550      1560
                                                CTGGCCTGCGTGGAGGTGCGACTGGCTCTCGAGGAGACAGGCACAGAGGTGAAAGAAGACGTGGT
CN235.seq(1>2269)                         →     ctggcctgcgtggaggtgcgactggctctcgaggagacaggcacagaggtgaaagaagacgtggt
CN235_1_(0708-108)NCDN-1_D11_11(7>925)    →     CTGGCCTGCGTGGAGGTGCGACTGGCTCTCGAGGAGACAGGCACAGAGGTGAAAGAAGACGTGGT

                                                    1570      1580      1590      1600      1610      1620
                                                AACTGCCTGCTATGCCCTTATGGAGTTGGGGATCCAGGAGTGTACCCGCTGTGAGCAGTCCCTGC
CN235.seq(1>2269)                         →     aactgcctgctatgcccttatggagttggggatccaggagtgtacccgctgtgagcagtccctgc
CN235_1_(0708-108)NCDN-1_D11_11(7>925)    →     AACTGCCTGCTATGCCCTTATGGAGTTGGGGATCCAGGAGTGTACCCGCTGTGAGCAGTCCCTGC

                                                1630      1640      1650      1660      1670      1680      1690
                                                TGAAGGAGCCCCAGAAAGTTCAGCTCGTGAGCATCATGAAAGAGGCCATCGGAGCTGTCATTCAC
CN235.seq(1>2269)                         →     tgaaggagccccagaaagttcagctcgtgagcatcatgaaagaggccatcggagctgtcattcac
CN235_1_(0708-108)NCDN-1_D11_11(7>925)    →     TGAAGGAGCCCCAGAAAGTTCAGCTCGTGAGCATCATGAAAGAGGCCATCGGAGCTGTCATTCAC
```

```
                                                      1700      1710      1720      1730      1740      1750
                                                TACCTGCTGCGGGTGGGGCCAGAGAAGCAGAAAGAGCCCTTTGTGTTTGCCTCTGTACGGATCCT
CN235.seq(1>2269)                          →    tacctgctgcgggtggggccagagaagcagaaagagccctttgtgtttgcctctgtacggatcct
CN235_1_(0708-108)NCDN-1_D11_11(7>925)     →    TACCTGCTGCGGGTGGGGCCAGAGAAGCAGAAAGAGCCCTTTGTGTTTGCCTCTGTACGGATCCT
CN235_1_(0708-109)NCDN-2_C11_1(20>1028)    →                                          CTTTGTGTTTGCCTCTGTACGGATCCT

                                                  1760      1770      1780      1790      1800      1810      1820
                                                GGGTGCCTGGCTGGCGGAGGAGACCTCATCCCTGCGTAAGGAGGTGTGCCAACTGCTGCCCTTCC
CN235.seq(1>2269)                          →    gggtgcctggctggcggaggagacctcatccctgcgtaaggaggtgtgccaactgctgcccttcc
CN235_1_(0708-108)NCDN-1_D11_11(7>925)     →    GGGTGCCTGGCTGGCGGAGGAGACCTCATCCCTGCGTAAGGAGGTGTGCCAACTGCTGCCCTTCC
CN235_1_(0708-109)NCDN-2_C11_1(20>1028)    →    GGGTGCCTGGCTGGCGGAGGAGACCTCATCCCTGCGTAAGGAGGTGTGCCAACTGCTGCCCTTCC

                                                      1830      1840      1850      1860      1870      1880
                                                TTGTCCGATATGCCAAGACACTCTATGAGGAGGCTGAGGAGG-CCAGTGACATTTCGCAGCAGGT
CN235.seq(1>2269)                          →    ttgtccgatatgccaagacactctatgaggaggctgaggagg-ccagtgacatttcgcagcaggt
CN235_1_(0708-108)NCDN-1_D11_11(7>925)     →    TTGTCCGATATGCCAAGACACTCTATGAGGAGGCTGAGGAGG-CCAGTGACATTTCGCAGCAG-T
CN235_1_(0708-109)NCDN-2_C11_1(20>1028)    →    TTGTCCGATATGCCAAGACACTCTATGAGGAGGCTGAGGAGG-CCAGTGACATTTCGCAGCAGGT

                                                  1890      1900      1910      1920      1930      1940      1950
                                                GGCTAACTTGGCC-ATCTCT-CCCACTACACCAGGGCCTGCTTGGCCAGGGGATGCTCTCC-GGC
CN235.seq(1>2269)                          →    ggctaacttggcc-atctct-cccactacaccagggcctgcttggccaggggatgctctcc-ggc
CN235_1_(0708-108)NCDN-1_D11_11(7>925)     →    GGCTAACTTGGCC-ATCTCT-CCCACTACACCAGGGCCTGCTTGGCCAGG
CN235_1_(0708-109)NCDN-2_C11_1(20>1028)    →    GGCTAACTTGGCC-ATCTCT-CCCACTACACCAGGGCCTGCTTGGCCAGGGGATGCTCTCC-GGC

                                                      1960      1970      1980      1990      2000      2010
                                                TCCTCCTTCCTGGCTGGTGCCACCTGACTGTTGAAGATGGTCCCCGGGAGATTTTGATCAAGGAA
CN235.seq(1>2269)                          →    tcctccttcctggctggtgccacctgactgttgaagatggtccccgggagattttgatcaaggaa
CN235_1_(0705-87)E1021-R_C01_11(7>925)     ←                GCTGGTGCCACCTGACTGTTGAAGATGGTCCCCGGGAGATTTTGATCAAGGAA
CN235_1_(0708-109)NCDN-2_C11_1(20>1028)    →    TCCTCCTTCCTGGCTGGTGCCACCTGACTGTTGAAGATGGTCCCCGGGAGATTTTGATCAAGGAA

                                                  2020      2030      2040      2050      2060      2070      2080
                                                GGAGCCCCCTCACTTCTGTGCAAGTACTTCCTGCAGCAGTGGGAACTCACATCCCCTGGCCATGA
CN235.seq(1>2269)                          →    ggagccccctcacttctgtgcaagtacttcctgcagcagtgggaactcacatcccctggccatga
CN235_1_(0705-87)E1021-R_C01_11(7>925)     ←    GGAGCCCCCTCACTTCTGTGCAAGTACTTCCTGCAGCAGTGGGAACTCACATCCCCTGGCCATGA
CN235_1_(0708-109)NCDN-2_C11_1(20>1028)    →    GGAGCCCCCTCACTTCTGTGCAAGTACTTCCTGCAGCAGTGGGAACTCACATCCCCTGGCCATGA

                                                      2090      2100      2110      2120      2130      2140
                                                TACCTCAGTGCTGCCAGACAGCGTGGAGATCGGCCTACAGACCTGTTGCCACATCTTCCTCAACC
CN235.seq(1>2269)                          →    tacctcagtgctgccagacagcgtggagatcggcctacagacctgttgccacatcttcctcaacc
CN235_1_(0705-87)E1021-R_C01_11(7>925)     ←    TACCTCAGTGCTGCCAGACAGCGTGGAGATCGGCCTACAGACCTGTTGCCACATCTTCCTCAACC
CN235_1_(0708-109)NCDN-2_C11_1(20>1028)    →    TACCTCAGTGCTGCCAGACAGCGTGGAGATCGGCCTACAGACCTGTTGCCACATCTTCCTCAACC

                                                  2150      2160      2170      2180      2190      2200      2210
                                                TGGTGGTCACCGCTCCCGGGCTGATCAAGCGCGACGCCTGCTTCACATCCCTTATGAACACCCTG
CN235.seq(1>2269)                          →    tggtggtcaccgctcccgggctgatcaagcgcgacgcctgcttcacatcccttatgaacaccctg
CN235_1_(0705-87)E1021-R_C01_11(7>925)     ←    TGGTGGTCACCGCTCCCGGGCTGATCAAGCGCGACGCCTGCTTCACATCCCTTATGAACACCCTG
CN235_1_(0708-109)NCDN-2_C11_1(20>1028)    →    TGGTGGTCACCGCTCCCGGGCTGATCAAGCGCGACGCCTGCTTCACATCCCTTATGAACACCCTG

                                                      2220      2230      2240      2250      2260      2270
                                                ATGACGTCACTGCCCTCACTAGTGCAGCAGCAAGGAAGACTGCTTCTAGCTGCCAACGTGGCCAC
CN235.seq(1>2269)                          →    atgacgtcactgccctcactagtgcagcagcaaggaagactgcttctagctgccaacgtggccac
CN235_1_(0705-87)E1021-R_C01_11(7>925)     ←    ATGACGTCACTGCCCTCACTAGTGCAGCAGCAAGGAAGACTGCTTCTAGCTGCCAACGTGGCCAC
CN235_1_(0708-109)NCDN-2_C11_1(20>1028)    →    ATGACGTCACTGCCCTCACTAGTGCAGCAGCAAGGAAGACTGCTTCTAGCTGCCAACGTGGCCAC

                                                  2280      2290      2300      2310      2320      2330      2340
                                                CTTGGGCCTCCTAATGGCCCGGCTCCTTAGCACCTCTCCAGCTCTCCAAGGAACTCCGGCCTCCC
CN235.seq(1>2269)                          →    cttgggcctcctaatggcccggctccttagcacctctccagctctccaaggaactccggcctccc
CN235_1_(0705-87)E1021-R_C01_11(7>925)     ←    CTTGGGCCTCCTAATGGCCCGGCTCCTTAGCACCTCTCCAGCTCTCCAAGGAACTCCGGCCTCCC
CN235_1_(0708-109)NCDN-2_C11_1(20>1028)    →    CTTGGGCCTCCTAATGGCCCGGCTCCTTAGCACCTCTCCAGCTCTCCAAGGAACTCCGGCCTCCC

                                                      2350      2360      2370      2380      2390      2400
                                                GAGGTTTCTTCGCAGCTGCCATCCTCTTTCTGTCACAGTCCCATGTGGCACGGGCCACCCCTGGC
CN235.seq(1>2269)                          →    gaggtttcttcgcagctgccatcctctttctgtcacagtcccatgtggcacgggccacccctggc
CN235_1_(0705-87)E1021-R_C01_11(7>925)     ←    GAGGTTTCTTCGCAGCTGCCATCCTCTTTCTGTCACAGTCCCATGTGGCACGGGCCACCCCTGGC
CN235_1_(0708-109)NCDN-2_C11_1(20>1028)    →    GAGGTTTCTTCGCAGCTGCCATCCTCTTTCTGTCACAGTCCCATGTGGCACGGGCCACCCCTGGC
```

```
                                                      2410      2420      2430      2440      2450      2460      2470
                                                      TCTGACCAGGCGGTGTTGGCCCTGTCCCCTGACTATGAGGGCATCTGGGCCGACTTGCAAGAGCT
CN235.seq(1>2269)                              →     tctgaccaggcggtgttggccctgtcccctgactatgagggcatctgggccgacttgcaagagct
CN235_1_(0705-87)E1021-R_C01_11(7>925)         ←     TCTGACCAGGCGGTGTTGGCCCTGTCCCCTGACTATGAGGGCATCTGGGCCGACTTGCAAGAGCT
CN235_1_(0708-109)NCDN-2_C11_1(20>1028)        →     TCTGACCAGGCGGTGTTGGCCCTGTCCCCTGACTATGAGGGCATCTGGGCCGACTTGCAAGAGCT

                                                           2480      2490      2500      2510      2520      2530
                                                      CTGGTTCTTGGGCATGCAGGCCTTCACAGGTTGTGTCCCTCTGTTGCCCTGGCTGGCCCCTGCCG
CN235.seq(1>2269)                              →     ctggttcttgggcatgcaggccttcacaggttgtgtccctctgttgccctggctggcccctgccg
CN235_1_(0705-87)E1021-R_C01_11(7>925)         ←     CTGGTTCTTGGGCATGCAGGCCTTCACAGGTTGTGTCCCTCTGTTGCCCTGGCTGGCCCCTGCCG
CN235_1_(0708-109)NCDN-2_C11_1(20>1028)        →     CTGGTTCTTGGGCATGCAGGCCTTCACAGGTTGTGTCCCTCTGTTGCCCTGGCTGGCCCCTGCCG

                                                      2540      2550      2560      2570      2580      2590      2600
                                                      CCCTGCGTTCCCGCTGGCCACAGGAGCTGCTACAGCTGCTAGGTAGTGTAAGCCCCAACTCTGTC
CN235.seq(1>2269)                              →     ccctgcgttcccgctggccacaggagctgctacagctgctaggtagtgtaagccccaactctgtc
CN235_1_(0705-87)E1021-R_C01_11(7>925)         ←     CCCTGCGTTCCCGCTGGCCACAGGAGCTGCTACAGCTGCTAGGTAGTGTAAGCCCCAACTCTGTC
CN235_1_(0708-109)NCDN-2_C11_1(20>1028)        →     CCCTGCGTTCCCGCTGGCCACAGGAGCTGCTACAGCTGCTAGGTAGTGTAAGCCCCAACTCTGTC

                                                           2610      2620      2630      2640      2650      2660
                                                      AAGCCCGAGATGGTGGCTGCCTACCAGGGCGTGCTCGTGGAATTGGCGCGGGCAAACCGGCTATG
CN235.seq(1>2269)                              →     aagcccgagatggtggctgcctaccagggcgtgctcgtggaattggcgcgggcaaaccggctatg
CN235_1_(0705-87)E1021-R_C01_11(7>925)         ←     AAGCCCGAGATGGTGGCTGCCTACCAGGGCGTGCTCGTGGAATTGGCGCGGGCAAACCGGCTATG
CN235_1_(0708-109)NCDN-2_C11_1(20>1028)        →     AAGCCCGAGATGGTGGCTGCCTACCAGGGCGTGCTCGTGGAATTGGCGCGGGCAAACCGGCTATG

                                                      2670      2680      2690      2700      2710      2720      2730
                                                      CCGGGAGGCCATGAGGCTGCAGGCGGGTGAAGAAACAGCCAGCCATTACCGCATGGCTGCTTTGG
CN235.seq(1>2269)                              →     ccgggaggccatgaggctgcaggcgggtgaagaaacagccagccattaccgcatggctgctttgg
CN235_1_(0705-87)E1021-R_C01_11(7>925)         ←     CCGGGAGGCCATGAGGCTGCAGGCGGGTGAAGAAACAGCCAGCCATTACCGCATGGCTGCTTTGG
CN235_1_(0708-109)NCDN-2_C11_1(20>1028)        →     CCGGGAGGCCATGAG-CTGCA-GCGGGTGAAGAAACAGCCAGCCATTACCGCATGGCTGCTTTGG

                                                           2740      2750      2760      2770      2780      2790
                                                      AGCAGTGCCTGTCAGAGCCCGACTACAAGGATGACGATGACAAGGATTACAAAGACGACGATGAT
CN235.seq(1>2269)                              →     agcagtgcctgtcagagcccgactacaaggatgacgatgacaaggattacaaagacgacgatgat
CN235_1_(0705-87)E1021-R_C01_11(7>925)         ←     AGCAGTGCCTGTCAGAGCCCGACTACAAGGATGACGATGACAAGGATTACAAAGACGACGATGAT
CN235_1_(0708-109)NCDN-2_C11_1(20>1028)        →     AGCAGTGCCTGTC

                                                      2800      2810      2820      2830      2840      2850      2860
                                                      AAGGACTATAAGGATGATGACGACAAATGAGAATTCGATATCAAGCTTATCGATAATCAACCTCT
CN235.seq(1>2269)                              →     aaggactataaggatgatgacgacaaatga
CN235_1_(0705-87)E1021-R_C01_11(7>925)         ←     AAGGACTATAAGGATGATGACGACAAATGAGAATTCGATATCAAGCTTATCGATAATCAACCTCT

                                                           2870      2880
                                                      GGATTACAAAATTTGGAAAGA
CN235_1_(0705-87)E1021-R_C01_11(7>925)         ←     GGATTACAAAATTTGGAAAGA
```

Supplement Figure 2: Sequence of AAV vector (Rat NCDN Expression AAV2/8)
